# Investigation of the Effect of Debris Position on the Detection Stability of a Magnetic Plug Sensor Based on Alternating Current Bridge

**DOI:** 10.3390/s24010055

**Published:** 2023-12-21

**Authors:** Siqi Zhang, Yucai Xie, Lianfeng Zhang, Yuwei Zhang, Shuyao Zhang, Chenzhao Bai, Wei Li

**Affiliations:** 1 College of International Collaboration, Dalian Maritime University, Dalian 116026, China; 2 Marine Engineering College, Dalian Maritime University, Dalian 116026, China; 3 Navigation College, Dalian Maritime University, Dalian 116026, China

**Keywords:** magnetic plug sensor, capture location, theoretical principle, finite element analysis

## Abstract

Magnetic plug-type abrasive particle sensors have a wide range of applications in oil detection, but there is little literature on the effect of abrasive particle position on detection accuracy. In this paper, an alternating current (AC) bridge-type abrasive particle detection sensor is designed, in which the sensing module utilizes permanent magnets to attract iron particles, and the induction coil is specially designed to detect the magnetic field fluctuation caused by iron particles. A corresponding model was also designed to evaluate the sensor’s sensitivity at different locations. In this paper, the magnetic field distribution of the sensor was first analyzed using finite element analysis software to obtain the magnetic field strength at different positions. Then, the response sensitivity of the sensor to particles and the effect of different positions on the detection results are explored through experiments. The simulation and the experimental results show substantial signal difference signal at different sensor positions. The method outlined in this article can determine the optimal sensing range for subsequent magnetic plug-type abrasive particle detection sensors and subsequently improve their reliability.

## 1. Introduction

In contemporary industry, lubricating oil reduces friction, facilitates power transmission, and dissipates heat in rotating machinery. The operational safety of marine vessels is compromised when wear debris accumulates in the lubrication system due to damage from rotating components [1]. Consequently, the characteristics of the debris in the oil provide a reliable indicator of the extent of wear [2]. The most relevant parameters when assessing wear particles are the size [3] and mass [4]. Throughout the operation of a mechanical system, the size and number of abrasive particles increases with wear. Numerous researchers have explored various techniques to detect minute particles (those with dimensions less than 100 μm) to signal the onset of abnormal wear preemptively. These methods include improved signal processing circuits [5], the use of magnetic media to generate high-gradient magnetic fields [6,7,8,9], the use of microfluidic channel detection [10,11], and the use of resonance in particle detection [12,13]. While these methodologies for early fault diagnosis are notably sensitive, their application is generally limited to examining individual particles, rendering them most appropriate for laboratory-based diagnostics and analyses performed using portable devices. However, as mechanical systems increasingly operate under high velocity and load, the deployment of online monitoring systems is essential for conditions equipment wear assessments. 

Magnetic sensors are fundamentally composed of a detection coil and a permanent magnet. Their ability to continuously capture and detect wear particles makes them exceptionally apt for online monitoring applications. Furthermore, Miller et al. [14] observed the presence of substantial particles (>250 μm) during the initial stages of wear, which were prevalent in scenarios of catastrophic wear. Dempsey et al. [15] found that mass measurements accurately indicate wear in gear fatigue test equipment. The researchers combined various techniques with magnetic sensors to detect the quality of the abrasive grains. These included optical sensors [16,17], resistive magnetic plug sensors [18], capacitive magnetic plug sensors [19], and inductive magnetic plug sensors [20]. There are two main optical detection methods: the light blockage method [16] and the imaging method [17]. Nonetheless, these techniques necessitate the implementation of intricate algorithms and high-performance hardware infrastructure. Sensors that combine an inductive coil with a permanent magnet are known for their robust resistance to interference and have been installed in aircraft engines [18]. Itomi et al. [19] designed a resistive magnetic plug sensor based on an eight-rod electrode-resistive bridge uniformly arranged around the permanent magnet, with a common electrode on the base surface that establishes a connection when iron particles accumulate on one side of the permanent magnet. Muthuvel et al. [20] introduced a capacitive magnetic plug sensor where the magnet not only attracted debris but also functioned as one of the sensor’s electrodes. In addition, electromagnetic technology is used in several areas of inspection. Imaz et al. [21] utilized magnetoelastic resonance to determine the viscosity of lubricating oils, finding a strong correlation between the resonance frequency and the viscosity of the oil as measured. Felix et al. [22] developed a prototype of a giant magnetic impedance sensor using a ferrogel and proposed an electrodynamic model describing magnetic impedance in multilayer films.

However, research addressing the impact of particle position on the detection sensitivity of sensors, which is crucial for improving sensor stability, is limited. 

This paper explores the effect of debris location on the stability of magnetic plug sensors, analyzes the inhomogeneity of the spatial magnetic field generated by the coil and the permanent magnet, and develops a coupled magnetic field model incorporating both components. Based on this model, how the different positions of metal particles affect the detection sensitivity of the sensor can be theoretically analyzed and simulated. By using an experimental setup built with AC bridges and measuring circuits, we can assess the sensor’s sensitivity and verify the effect of particle position on the stability of the sensor.

## 2. Sensor Structure

**Figure 1:**
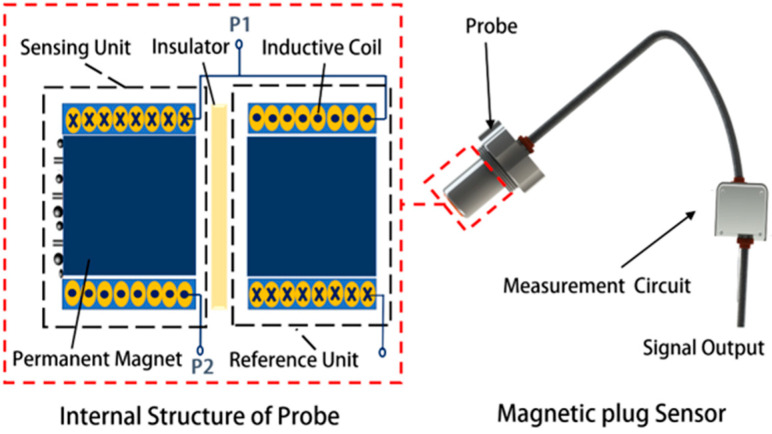
Schematic of magnetic plug sensor, P1 is the series output endpoint of the two coils and P2 is one of the inputs of the coils.

**Figure 2:**
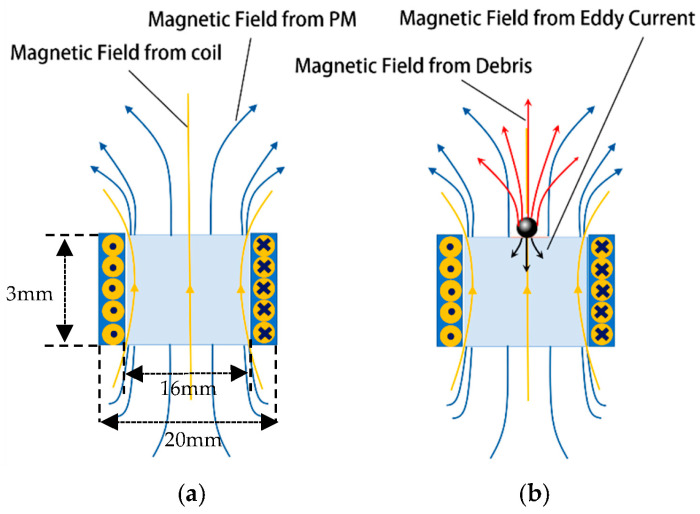
Schematic of magnetic field change in sensing unit: (**a**) No debris, the magnetic field is generated by magnetic and a coil. (**b**) Ferromagnetic wear debris, where the debris causes the magnetic field to change.

As shown in Figure 1, the designed magnetic plug sensor mainly includes a probe and a measurement circuit. The sensing unit and reference unit are inside the probe. Based on an inductive AC bridge circuit, they each serve as one of the bridge arms. Other electronic components are arranged in the measurement circuit. The detecting coil will form an alternating magnetic field when AC power is applied, which is superimposed with the magnetic field of the permanent magnet as the detecting source field, as shown in Figure 2a. When the permanent magnet adsorbs the ferromagnetic metal particles, the ferromagnetic metal particles will be subject to magnetization and the eddy current effect, and magnetization takes the dominant role because the ferromagnetic metal particles have high magnetic permeability. Little eddy current effects are generated inside the ferromagnetic metal particles in the alternating magnetic field, and their direction of action is always opposite to the direction of the magnetic field of the coil, as shown in Figure 2b. The ferromagnetic metal particles affect the magnetic field fluctuations, which causes the coil’s impedance to change. Due to the uneven distribution of the alternating magnetic field generated by the coil and the magnetic field of the permanent magnet, the degree of magnetization and the size of the eddy currents formed by the ferromagnetic metal particles are different at different positions. The resulting changes in the coil impedance induced by the same ferromagnetic metal particles at different positions are different, which will lead to detection errors. Therefore, it is necessary to analyze the magnetic field distribution of the detection sensor from the detection mechanism and study the influence of the structure of the detection unit on the performance of the magnetic plug abrasive particle sensor.

## 3. Modeling and Simulation

### 3.1. Theoretical Analysis

**Figure 3:**
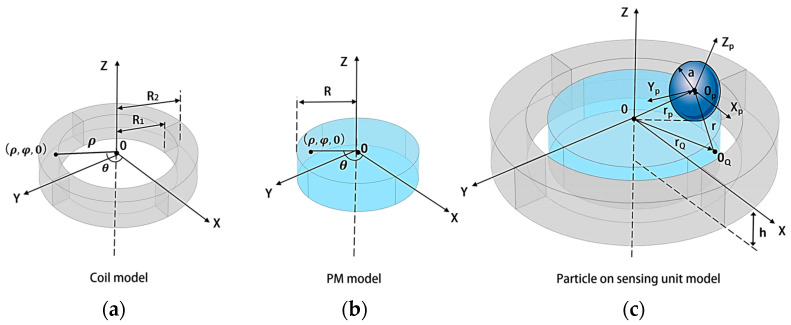
Coordinate system on sensing unit: (**a**) the model of the coil, (**b**) the model of the permanent magnet, and (**c**) the model of the particle on sensing unit model.

Our team previously investigated the effect of the spatial position of metal particles on the variations in coil inductance [23]. In this paper, we further analyze the magnetic field of a sensing unit consisting of a coil and a magnet. As shown in Figure 3, an orthogonal coordinate system (*X_p_*, *Y_p_*, *Z_p_*) is established, with the center of the particle as the origin. The iron particle is magnetized by magnetic field *B*, and *Z_p_* is parallel to *B*. The particle located at point *P* causes a magnetic vector potential change in the *Q* location, as follows:

(1) $$\Delta A\left(Q\right)=\frac{1}{4\pi }v\chi_{a}B_{}\times \frac{r_{Q}-r_{P}}{{\left|r_{Q}-r_{P}\right|}^{3}}.$$

*ν* is particle volume. *χ_a_* is the magnetic susceptibility of iron particles in the time-harmonic magnetic field:

(2) $$\chi_{a}=\frac{3}{2}\frac{\left(a^{3}k^{2}+2\mu_{r}-1)\sin(ak)+ak(2\mu_{r}+1)\cos(ak\right)}{\left(a^{3}k^{2}+\mu_{r}-1)\sin(ak)-ak(\mu_{r}-1)\cos(ak\right)}.$$

The coil impedance change is as follows:

(3) $$\Delta \tilde{Z}=j\omega n_{c}\int_{0}^{2\pi }d_{\varphi_{Q}}\int_{R_{1}}^{R_{2}}\rho_{Q}d\rho_{Q}\int_{-h}^{0}\left[\frac{\Delta A(Q)}{I}\right]dz_{Q}.$$

The coil inductance change is as follows:

(4) $$\Delta L=\frac{I_{m}(\Delta \tilde{Z})}{\omega }$$

The position of the particles on the permanent magnet affects the inductance of the coil. This is mainly caused by the magnetic properties proposed by the uneven distribution of the magnetic field strength at different coil positions, as shown in Equation (1). When studying the effect of the same particle at different positions on the coil inductance, this is only related to the radial position ρ of the particle. The change in the coil’s inductance at ρ = *R* is more significant than the change in inductance at ρ = 0.

### 3.2. Finite Element Analysis

**Figure 4:**
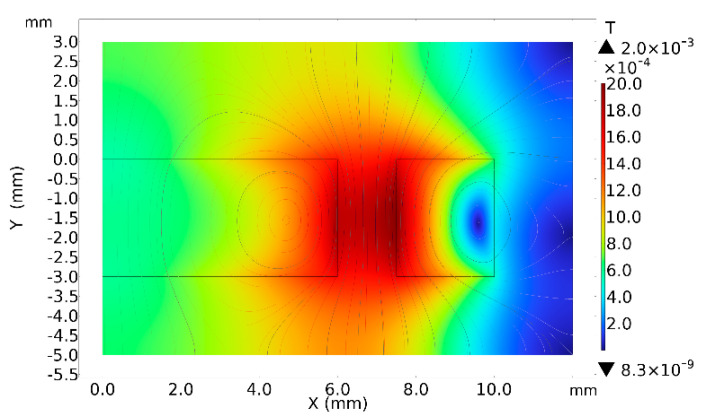
Distribution of flux density patterns of sensing units at cross-section “Y = 0, X > 0”.

Before conducting the experiments, the magnetic field distribution of the pairs of sensors was first simulated using the COMSOL Multiphysics 5.4 finite-element analysis software to explore the relationship between the magnetic field and the change in position. The geometric model in the finite element analysis is consistent with Figure 3. Due to the symmetrical structure of the sensing unit, its flux density mode at the cross-section “Y = 0, X > 0” is shown in Figure 4. When the excitation signal of 10 V 100 kHz is applied to the sensor, the coil generates an alternating magnetic field, and the magnetic field density mode is the largest in the contact area between the coil and the magnet because the magnetic field at this position is the sum of the magnetic field of the coil and the magnet, which are superimposed on each other. Therefore, the magnetic field is higher than this location.

**Figure 5:**
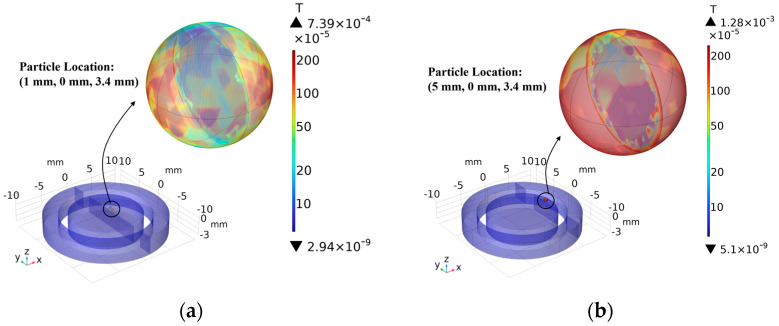
Effect of particle position on magnetic flux density: (**a**) particle position (1 mm, 0 mm, and 3.4 mm) and (**b**) particle position (5 mm, 0 mm, and 3.4 mm).

The amount of inductance change caused by the same particle at different locations is explored using parametric simulations. As shown in Figure 5, an iron particle with a particle size of 400 μm is selected and controlled so that it occurs at different positions on the surface of the detection unit. It can be seen from the figure that the flux density more significant at the intersection of the magnet and the coils, compared to the center position, which is attributed to the fact that the strength of the magnetic field at this position is stronger than that at other positions. 

The particles were selected to move along the X-axis direction, at this time Y = 0 mm, Z = 3.4 mm because the shape of the sensor is circular, as shown in Figure 5, so the change along the Y-axis direction is consistent with the change along the X-axis direction. The coil inductance change caused by the particles in different positions is shown in Table 1, and it can be clearly seen that the signal in the middle position is significantly weaker than that in the edge position. Therefore, when designing high-precision sensors, the influence of the position on the detection results should be fully considered, and the detection accuracy can be improved by controlling the position of the particle adsorption.

## 4. Experimental Validation

### 4.1. Experiment System

**Figure 6:**
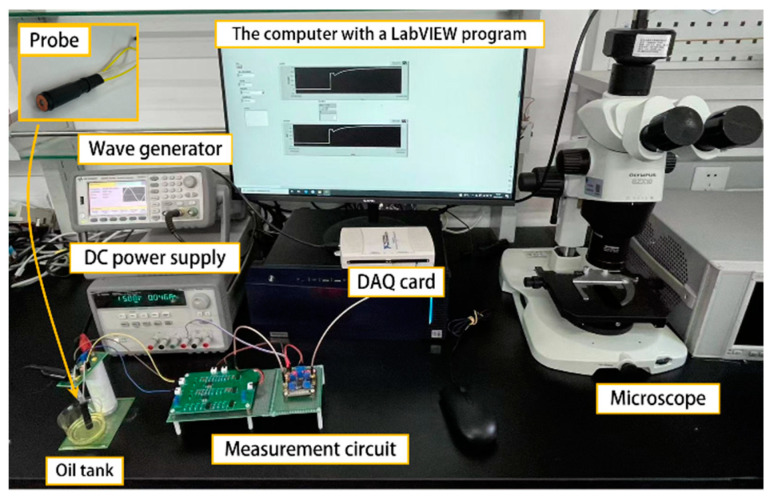
Photograph of the experimental system.

The experimental bench is shown in Figure 6. The experimental bench consists of a sensing unit, a waveform generator (KEYSIGHT 33600A Series, KEYSIGHT, Santa Rosa, CA, USA), an oscilloscope (Agilent Technologies MS071048, Agilent, Santa Clara, CA, USA), a microscope (Olympus Corporation SZX2-ZB10, Olympus Corporation, Tokyo, Japan), a DC power supply (Agilent E3631A, Agilent, USA), signal conditioning circuits, a computer with a LabView data acquisition unit (LabVIEW, National Instruments, Austin, TX, USA), and a data acquisition card (NI USB_6211, National Instruments, Austin, TX, USA). The waveform generator produces an AC excitation signal to drive the sensing unit, and the initial output signal of the sensor is an AC signal, which is not conducive to the extraction and collection of information; therefore, the signal is processed through the amplification circuit and filtering circuit to be converted into a DC signal, which is then read by the data acquisition card, the adsorption of particles is observed through a microscope, and the DC power supply provides power for the signal processing circuit.

**Figure 7:**
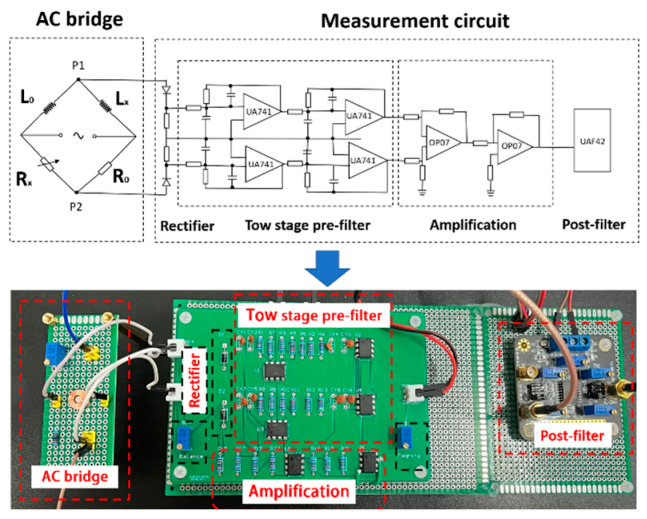
Circuit structure.

The measurement circuit is used to enhance and process the output signals of the sensor, as shown in Figure 7. The AC bridge is used to measure the inductance values *L_x_* and *L*_0_. They are converted into voltage signals and processed by the measurement circuit. The AC voltages (*V_x_* and *V*_0_) are rectified into pulsating DC signals by a half-wave rectifier circuit. The pulsating DC signal is filtered to DC by a pre-filter consisting of two filter stages. The difference (*V_x_* − *V*_0_) is re-accepted by the differential amplifier circuit, which is the first stage of amplification. It is further amplified by a second stage of amplification. To further improve the signal-to-noise ratio, a post-filter is required. Theoretically, *R_x_* and *R*_0_ are equal, so the AC bridge is balanced before testing. In practice, however, the environment affects the coil inductance, *L*_0_, and *L_x_*, especially the temperature. Therefore, the potentiometer (*R_x_*) is set to adjust the balance of the AC bridge.

In this experiment, the neodymium magnet has a diameter of 12 mm and a height of 3 mm. The dimensions of the coil’s dimensions were 20 mm in diameter and 3 mm in height. Metal abrasive particles are iron particles with a particle size of 250 μm~350 μm, which are prepared in six different concentrations, and the excitation signal is 10 V 100 kHz. The sensor focuses on detecting mass and, therefore, does not differentiate between the size and shape of the particles.

### 4.2. Sensitivity Test

**Figure 8:**
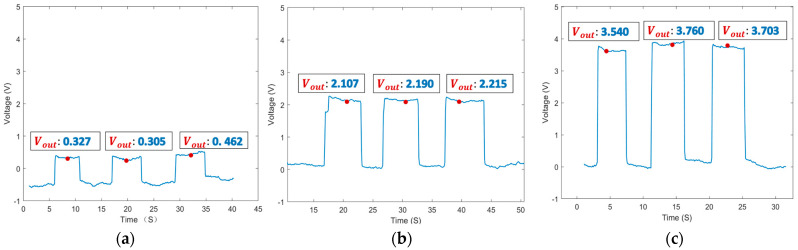
Experimental output results (*V_out_*): (**a**) voltage detection results for 0.11 mg of iron particles, (**b**) voltage detection results for 0.33 mg iron particles, and (**c**) voltage detection results for 0.55 mg iron particles.

The prepared experimental samples were placed on the sensing surface of the sensor, respectively, to obtain the induced voltage caused by the particles, and some of their outputs are shown in Figure 8. It can be seen in the figure that the particles were subjected to multiple detections with some differences in the signals, which is due to the fact that the position of the particles on the sensing surface changes when they are adsorbed, and therefore the output signals are different. The peck signals’ duration corresponds to the particles’ adhesion time of the particles, and the amplitude of the signal corresponds to the quality of the adhered particles. When analyzing the signal concentration, the particle quantity is judged based on the signal’s amplitude, so changes in the base value of the signal do not affect the detection results. 

**Figure 9:**
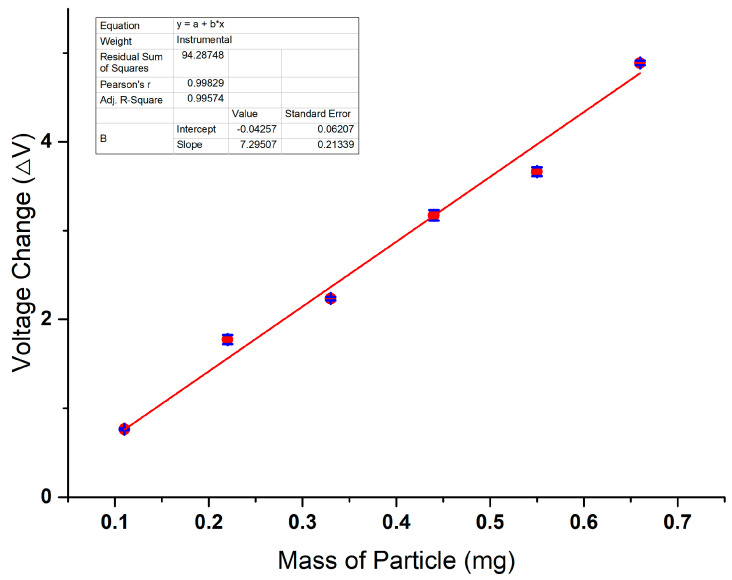
Sensor response of particles with different mass.

The particle-induced voltage change (Δ*V*) in the sensor is shown in Figure 9. Compared to the *V_out_* value, the Δ*V* value is unaffected by the fundamental voltage fluctuations and provides a more accurate representation of the particle mass. The error bars are derived from the data of three replicate experiments. There is a positive correlation between the particle mass and the voltage signal.

### 4.3. Stability Test

**Figure 10:**
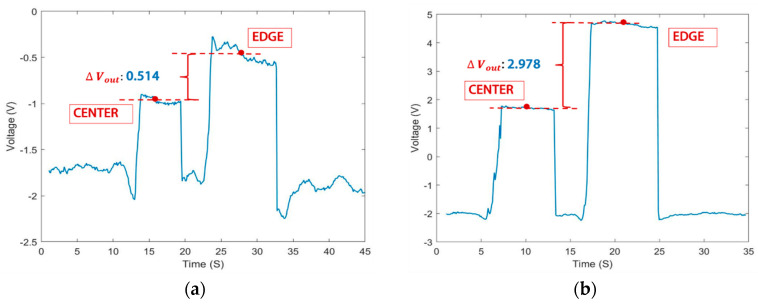
Particles with the same mass captured in the center detection region or edge detection region: (**a**) voltage signal variation at different positions for 0.22 mg particles and (**b**) voltage signal variation at different positions for 0.55 mg particles.

The experimental manipulation controlled the attraction of metal particles to different regions (center or edge) of the sensor. The voltage signal output from the sensor is shown in Figure 10. Different particle positions result in differences in voltage output (Δ*V_out_*). Figure 10a shows that the voltage signal at the edge of a 0.22 mg particle is 0.514 volts, which is greater compared to when the particle is at the center. In Figure 10b, 0.55 mg of particles at different locations results in a voltage change of 2.978 volts. The results show that the edge detection region of the sensor is more sensitive than the center detection region, and the larger the particle size, the greater the change in signal. The main reason for this phenomenon is that the magnetic field density at the edge of the probe is much higher than that at the center. Therefore, when analyzing the particle concentration, the difference in the captured particle position will lead to signal deviations, which also indicates that the magnetic field inhomogeneity and the reasonableness of the layout of the detection position should be fully considered when carrying out the sensor design.

## 5. Discussion

The article presents a simulation and experimental investigation of the position error of the magnetic plug-type abrasive particle detection sensor. It can be seen from Table 1 that the inductance of the particles changes by 13.2 μH when the position of the particles in the coil changes by 4 mm along the X-axis. From the experimental results, it can be seen that the change in the voltage of the iron particles of 0.55 mg at the center is 2.978 V smaller than that produced at the edges, and the difference between the signals is very evident. The simulation and experimental results show that the particle position will have an impact on the experimental results. The main reason for this result is the uneven distribution of the magnetic field of the coil. In addition, the static magnetic field generated by the magnet will be superimposed with the alternating magnetic field generated by the coil, and the static magnetic field at the edge of the magnetic field strength is greater than that of the center position; this phenomenon further exacerbates the impact of the adsorption position of the particles on the results of the detection, which further aggravates the influence of the particle adsorption position on the detection results, thus making the detection effect at the edge better than that at the center.

## 6. Conclusions

In this paper, the effect of particle position on the magnetic plug sensor is investigated with respect to the position error problem of the magnetic plug particle detection sensor. The simulation results show that the output signals of particles with different positions significantly differ due to the existence of a hole in the center, resulting in different magnetic field strengths at the edge of the coil and at the hole, which have different magnetization effects on the particles, thus resulting in detection errors. The experiment also verifies the phenomenon that the sensitivity is strongest when the particle is close to the edge of the magnet and coil and weaker when the particle is close to the center of the proposed magnet. First, the gap between the outside of the magnet and the inside of the coil should be as small as possible to utilize the highest magnetic field in that region. Second, the height of the magnet should be lower than the height of the coil to prevent the uneven magnetization of wear debris. Third, a ring-shaped proposed magnet is used instead of a cylindrical permanent magnet to ensure that particles are captured in the higher magnetic field. This study provides a new reference method for the design of magnetic plug-type abrasive grain sensors, which could help to improve sensor accuracy.

## Figures and Tables

**Table 1:** The relationship between the particle location and the coil inductance.

Position of the particle in the X-axis (mm)	1	2	3	4	5
Detection coil inductance (μH)	4558.3	4561.0	4565.9	4571.5	4571.5

## Data Availability

Data are contained within the article.
